# One‐year trends from the LANDMARC trial: A 3‐year, pan‐India, prospective, longitudinal study on the management and real‐world outcomes of type 2 diabetes mellitus

**DOI:** 10.1002/edm2.316

**Published:** 2021-12-01

**Authors:** Ashok K. Das, Sanjay Kalra, Shashank Joshi, Ambrish Mithal, Prasanna Kumar K. M., Ambika G. Unnikrishnan, Hemant Thacker, Bipin Sethi, Subhankar Chowdhury, Romik Ghosh, Sukanya Krishnan, Arjun Nair, Senthilnathan Mohanasundaram, Shalini K. Menon, Vaibhav Salvi, Deepa Chodankar, Saket Thaker, Chirag Trivedi, Subhash K. Wangnoo, Abdul H. Zargar, Nadeem Rais

**Affiliations:** ^1^ Pondicherry Institute of Medical Sciences Puducherry India; ^2^ Bharti Hospital Karnal India; ^3^ Lilavati Hospital Mumbai India; ^4^ Medanta‐The Medicity Gurgaon India; ^5^ Centre for Diabetes and Endocrine Care Bengaluru India; ^6^ Chellaram Diabetes Institute Pune India; ^7^ Bhatia Hospital Mumbai India; ^8^ Care Hospital Hyderabad India; ^9^ IPGME and R and SSKM Hospital Kolkata India; ^10^ Medical Affairs Sanofi Mumbai India; ^11^ Clinical Study Unit Sanofi Mumbai India; ^12^ Apollo Hospital Education and Research Foundation New Delhi India; ^13^ Center for Diabetes and Endocrine Care Srinagar India; ^14^ Chowpatti Medical Centre Mumbai India

**Keywords:** diabetes mellitus, glycaemic control, India, real‐world outcomes

## Abstract

**Introduction:**

Longitudinal data on management and progression of type 2 diabetes mellitus (T2DM) in India are scarce. LANDMARC (CTRI/2017/05/008452), first‐of‐its‐kind, pan‐India, prospective, observational study aimed to evaluate real‐world patterns and management of T2DM over 3 years.

**Methods:**

Adults (≥25 to ≤60 years old at T2DM diagnosis; diabetes duration ≥2 years at enrolment; controlled/uncontrolled on ≥2 anti‐diabetic agents) were enrolled. The first‐year trends for glycaemic control, therapy and diabetic complications, including those from metropolitan and non‐metropolitan cities are reported here.

**Results:**

Of 6236 enrolled participants, 5654 completed 1 year in the study. Although the overall mean glycated haemoglobin (HbA1c) improved by 0.5% (baseline: 8.1%) at 1 year, only 20% of the participants achieved HbA1c <7%. Participants from metropolitan and non‐ metropolitan cities showed similar decrease in glycaemic levels (mean change in HbA1c: −0.5% vs. −0.5%; *p* = .8613). Among diabetic complications, neuropathy was the predominant complication (815/6236, 13.1% participants). Microvascular complications (neuropathy, nephropathy and retinopathy) were significantly (*p* < .0001) higher in non‐metropolitan than metropolitan cities. Hypertension (2623/6236, 78.2%) and dyslipidaemia (1696/6236, 50.6%) continued to be the most commonly reported cardiovascular risks at 1 year. After 1 year, majority of the participants were taking only oral anti‐diabetic drugs (OADs) (baseline: 4642/6236 [74.4%]; 1 year: 4045/6013 [67.3%]), while the proportion of those taking insulin along with OADs increased (baseline: 1498/6236 [24.0%] vs. 1 year: 1844/6013 [30.7%]). Biguanides and sulfonylureas were the most used OADs. The highest increase in use was seen for dipeptidyl peptidase‐IV inhibitors (baseline: 3047/6236 [48.9%]; 1 year: 3529/6013 [58.7%]). Improvement in all glycaemic parameters was significantly (*p* < .0001) higher in the insulin vs. the insulin‐naïve subgroups; in the insulin‐naïve subgroup, no statistical difference was noted in those who received >3 vs. ≤3 OADs.

**Conclusions:**

First‐year trends of the LANDMARC study offer insights into real‐world disease progression, suggesting the need for controlling risk factors and timely treatment intensification in people with T2DM.

## Graphical abstract

The literature describing the burden of T2DM in India are sparse. The present study reports 1‐year trend from LANDMARC study, a nationwide, prospective, longitudinal study planned for 3 years. This study highlights some important observations in terms of diabetes complications, treatment patterns and glycaemic control in T2DM patients from India and also emphasizes the disease burden in metropolitan and non‐metropolitan cities. This study revealed that the burden of uncontrolled T2DM is high in India with only 20% of participants achieving glycaemic control in 1 year (HbA1c <7%; 53  mmol/mol).

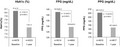

## 1 INTRODUCTION

Diabetes mellitus is rising at an alarming rate worldwide. As per 2019 International Diabetes Federation (IDF) estimates, 77 million people were living with diabetes in India in 2019.^1^ With an age‐adjusted comparative prevalence of 10.4% in the age group of 20–79 years, India has become a major diabetes centre in Southeastern Asia.^1^, ^2^, ^3^ The Indian Council of Medical Research‐INdia DIABetes (ICMR–INDIAB) study reports an overall diabetes prevalence of 7.3% across 15 states of India. The prevalence in urban areas is two times higher than that in rural areas (11.2% vs. 5.2%, respectively).^4^ Nearly, 90% of the overall diabetes burden is due to type 2 diabetes mellitus (T2DM) cases owing to rapid global urbanization, ageing and obesity, thus promoting the proliferation of the disease either via genetic inheritance or external factors.^1^

India carries a huge burden of T2DM largely due to the ‘thin outside‐fat inside’ phenotype.^5^, ^6^, ^7^ This feature augments insulin resistance and onset of T2DM at an early age, which accelerates the risk of microvascular and macrovascular complications and ultimately increases the morbidity and mortality rates associated with these complications.^8^, ^9^, ^10^, ^11^ Diabetes is often diagnosed late in India and worsened by vascular complications in the form of metabolic abnormalities and angiopathies.^8^, ^12^, ^13^ Indians have an inherent tendency of acquiring cardiovascular (CV) risk (hypertension, dyslipidaemia and albuminuria).^14^ The presence of both hypertension and dyslipidaemia in people with diabetes has additive adverse impact on the vascular endothelium, which substantially accelerates the risk of microvascular and macrovascular complications such as neuropathy, nephropathy, retinopathy, coronary heart disease and stroke.^15^, ^16^, ^17^

Although glycaemic indices are the primary focus of diabetes management, several factors such as age, body mass index (BMI), high blood pressure, presence of chronic kidney disease, cholesterol and triglyceride levels, duration of diabetes and family history of CV disease influence treatment outcomes. Considering all these factors, a patient‐centric approach is of utmost importance to achieve glycaemic targets.^18^, ^19^, ^20^ In addition, early initiation of favourable pharmacotherapy and sustained glycaemic control along with lifestyle change is a well‐known treatment strategy in preventing/delaying vascular complications of diabetes.^19^, ^20^, ^21^ Overcoming clinical inertia by early initiation of a combination therapy or insulin therapy could help in achieving glycaemic targets faster in people who are poorly controlled on monotherapy and thus alleviate the burden of diabetes‐related vascular complications.^21^, ^22^

Comprehensive, robust, longitudinal and long duration data on glycaemic, therapy and diabetic complication trends in people with diabetes living in different regions of India (including metropolitan and non‐metropolitan cities) are unavailable. Such data could uncover the challenges hampering diabetes care and control in India. Real‐world evidence can provide better insights into therapy patterns over time, drug adherence and the course of diabetic complications. These data will also help to assess the benefits of optimal treatment in preventing complications, and effects of changing or customizing medications for the existing diabetes condition. The LongitudinAl Nationwide stuDy on Management And Real‐world outComes of diabetes in India (LANDMARC) is first‐of‐its‐kind national, prospective, multicentre, observational study conducted in participants with T2DM from India to understand the treatment patterns, glycaemic control and diabetes complications in a real‐world setting, over a period of 3 years. The study protocol^23^ and baseline data^24^ have been published earlier. The aim of this 1‐year data analysis is to understand the longitudinal trends in glycaemic control, treatment pattern, CV risk and diabetic complications in adult Indian participants with T2DM. In addition, this 1‐year data analysis compares the glycaemic status of people with T2DM in metropolitan and non‐metropolitan cities of India.

## 2 MATERIALS AND METHODS

### 2.1 Study design

The evaluation period of this multicentre, prospective, observational study was 36 months (March 2017–March 2021), which was divided into seven visits with an interval of 6 months. The results in this manuscript represent the first year (within a window period of −90 days or +45 days) of the 3‐year evaluation period.^23^

At visit 3, the endpoints assessed were the proportion of participants with macrovascular complications (CV disease [CVD] and peripheral vascular disease [PVD]), microvascular complications (retinopathy, nephropathy and neuropathy), CV risk factors (hypertension, dyslipidaemia and albuminuria) and frequency/severity of hypoglycaemia episodes. The proportion of participants taking oral anti‐diabetic drugs (OADs) and injectable glucose‐lowering drugs were also assessed. Data related to anthropometry (weight) and glycaemic control status (fasting plasma glucose [FPG], post‐prandial glucose [PPG] and glycated haemoglobin [HbA1c]) were collected. The glycaemic parameters and the complications amongst participants from metropolitan and non‐metropolitan sites were also assessed.

### 2.2 Study participants

People who were ≥25 and ≤60 years of age at the time of T2DM diagnosis were recruited. Eligible participants were those with T2DM for at least 2 years at the time of enrolment and were controlled/uncontrolled on ≥2 anti‐diabetic agents. Participants who had known type 1 diabetes mellitus (T1DM) and secondary diabetes (eg gestational diabetes and fibrocalculus pancreatic diabetes) and those who had limited life expectancy due to terminal diseases were not included in the study. The details of the study design, methodology, inclusion/exclusion criteria and statistical analysis have been published previously.^23^, ^24^

The protocol complies with the Declaration of Helsinki and this study is conducted in accordance with the principles laid by the 18th World Medical Assembly (Helsinki, 1964) and all subsequent amendments. The study is also in accordance with the guidelines for Good Epidemiology Practice [US & European]^25^, ^26^ and is aligned with the local regulations, ethics committee(s) (institutional review board/independent ethics committee) and competent authorities. The study was approved by the ethics committees of all participating sites (or a central ethics committee where applicable). All the participants provided written informed consent before data collection/documentation.

### 2.3 Selection of investigators

Investigators (general practitioners, endocrinologists and diabetologists) who were willing to participate were selected based on the requisite qualification, facilities and resources to conduct this study. The selected 450 sites represent the 4 geographical regions (East, West, North and South), urban/rural practice, clinic/hospital bases and government/corporate hospital/nursing homes across India.

### 2.4 Data collection

Information related to study endpoints was collected prospectively every six months up to the end of the study at 36 months. The study design was planned to mirror real‐life management of participants with T2DM; therefore, no assessments were mandated, and the available data were recorded in electronic‐Case Report Forms (e‐CRFs). Data quality control was performed by qualified designated personnel. Any adverse drug reaction related to any Sanofi product (clinical signs, laboratory values or other) were reported and followed up until the clinical recovery was complete and laboratory results (if clinically significant) had returned to normal, or until progression had been stabilized. This was a planned interim analysis to assess changes in the disease characteristics from baseline and may need modification in the assessment parameters for subsequent interim analyses and the final analysis.

## 3 RESULTS

### 3.1 Demographics and baseline characteristics

**FIGURE 1:**
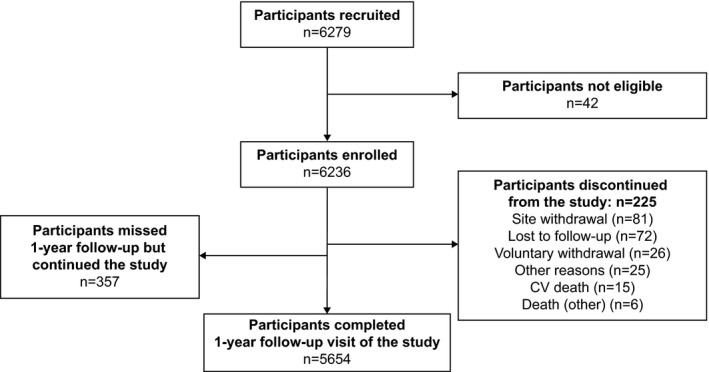
Participants disposition. *n* = number of participants

Of the 6236 eligible participants enrolled in this study, 5654 completed one year in the study (Figure 1). Details of demographics and baseline characteristics have been published earlier.^24^ At baseline, the mean (standard deviation [SD]) age of the participants was 52.1 (9.2) years with 57.0% (3553/6236) of the study population in the age range of 50–65 years; more than half of the participants (3528/6236, 56.6%) were men. The mean (SD) baseline BMI was 27.2 (4.6) kg/m^2^, and majority of participants were obese (4150/6217, 66.8%). At baseline, the mean (SD) duration of diabetes was 8.6 (5.6) years; duration of diabetes was longer in the insulin‐treated participants compared with insulin‐naïve participants (mean [SD]: 11.3 [6.6] and 7.7 [5.0] years, respectively). Most participants (74.4%, 4642/6236) were taking only OADs; while, 24.0% (1498/6236) were taking OADs + insulin; 0.7% (45/6236) were receiving OADs + non‐insulin injectable glucose‐lowering drugs; 0.4% (26/6236) were using insulin alone; and 0.4% (25/6236) of the participants were on OADs + insulin + non‐insulin injectable glucose‐lowering drugs (Table S1).

### 3.2 Microvascular and macrovascular complications at the end of the first year

At 1 year, the most frequently reported microvascular complication was neuropathy. It was reported in 815/6236 participants (13.1%), while nephropathy was reported in 180/6236 participants (2.9%) and retinopathy in 152/6236 participants (2.4%) (Table 1A). Overall, 42 new cases of microvascular complications were reported in 41 participants in 1 year. Notably, neuropathy was the most common (29 cases), followed by nephropathy (7 cases) and retinopathy (6 cases) (Table 1A). At 1 year, retinopathy was significantly higher in the HbA1c ≥7% subgroup (*p* = .0115), BMI ≥23 kg/m^2^ subgroup (*p* = .0035), and in those with CV risk factors (*p* < .0001) (Table S2).

**TABLE 1:** Proportion of participants with (A) microvascular and (B) macrovascular complications at baseline and at 1 year (*N* = 6236)

	Baseline *n* (%)	1 year *n* (%)	Participants with new complications 1 year *n*
(A) Microvascular complications			
Neuropathy	737 (11.8)	815 (13.1)	29
Nephropathy	154 (2.5)	180 (2.9)	7
Retinopathy	141 (2.3)	152 (2.4)	6
(B) Macrovascular complications			
Acute coronary syndrome^a^	92 (1.5)	95 (1.5)	3
Myocardial infarction^b^	74 (1.2)	78 (1.3)	4
Peripheral vascular disease^b^	45 (0.7)	55 (0.9)	11
Stroke^b^	30 (0.5)	32 (0.5)	2

Note

Values are presented as *n* (%) unless specified otherwise. Newly documented and pre‐existing complications were reported as incidence and prevalence, respectively. This is an interim analysis and possible modifications on variables and data could be performed for the subsequent interim analyses and final analysis.

Abbreviations: *N*, number of participants analysed; *n*, number of participants with non‐missing results at the visit.

^a^ Complications are part of the definition for the secondary endpoint.

^b^ Complications are part of the definition for the primary endpoint.

At 1 year, among the newly reported cases of macrovascular complication, cases of PVD were reported by 11 participants, myocardial infarction by 4 participants, acute coronary syndrome (ACS) by 3 participants and stroke by 2 participants (Table 1B). At the end of 1 year, a total of 21 deaths were reported, of which, 15 deaths were attributed to CV causes (myocardial infarction [*n* = 7], sudden death [*n* = 6], stroke and coronary artery procedure [*n* = 1, each]). Occurrence of each of the macrovascular complications (PVD, myocardial infarction, ACS and stroke) did not differ greatly between the HbA1c ≥7% and <7% subgroups, BMI ≥23 and <23 kg/m^2^ subgroups, and participants with and without CV risk (Table S2).

**FIGURE 2:**
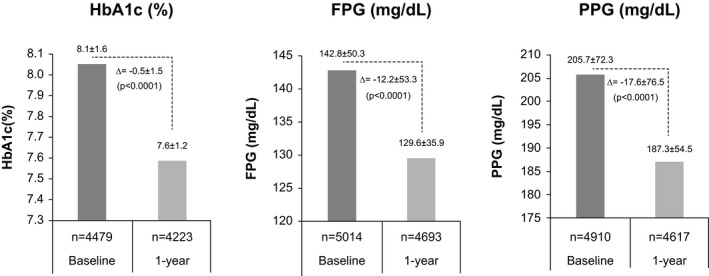
Change in glycaemic parameters at the end of 1 year. Values are presented as mean ± standard deviation. FPG, fasting plasma glucose; HbA1c, glycated haemoglobin; *n*, number of participants analysed; PPG, postprandial glucose

Although the mean (SD) HbA1c improved by 0.5% (1.5) (baseline: 8.1% [1.6]) at the end of 1 year, there was an overall increase in the number of participants with microvascular and macrovascular complications (Table 1A,B and Figure 2).

### 3.3 Cardiovascular risk factors

Nearly half of the participants had CV risk at baseline (3281/6236, 52.6%), with a marginal increase noted at 1 year (3355/6236; 53.8%). Of the 55 new cases of CV risk factors, dyslipidaemia (28 cases) and hypertension (25 cases) were the most commonly reported risk factors followed by albuminuria (2 cases) (Table 2). Hypertension and dyslipidaemia were more common among participants with BMI ≥23 kg/m^2^ than in those with BMI <23 kg/m^2^ (*p* < .0001 and *p* = .0023, respectively). Hypertension was reported in more men than women at 1 year (*p* = .0032) (Table 2).

**TABLE 2:** Summary of CV risk factors at 1 year by HbA1c, BMI and gender (*N* = 6236)

CV risk factors	Total *N* = 6236		
	Baseline	1 year	Participants with new CV risk factors at 1 year
Total number of CV risk factors, Ne	4419	4547	55
Participants with CV risk factors	3281 (52.6)	3355 (53.8)	54
Hypertension^b^	2566 (78.2)	2623 (78.2)	25
Dyslipidaemia^b^	1635 (49.8)	1696 (50.6)	28
Albuminuria^b^	153 (4.7)	160 (4.8)	2
Family History of PCD^b^	65 (2.0)	65 (1.9)	–
No complications	2564	–	–
Unknown^c^	391	–	–

Risk factors	HbA1c <7% *n* (%)	HbA1c ≥7% *n* (%)	*p*‐Value^a^	BMI <23 kg/m^2^ *n* (%)	BMI ≥23 kg/m^2^ *n* (%)	*p*‐Value^a^	Men *n* (%)	Women *n* (%)	*p*‐Value^a^
Hypertension	511 (8.2)	1259 (20.2)	.3775	297 (4.8)	2017 (32.3)	<.0001	1427 (22.9)	1196 (19.2)	.0032
Dyslipidaemia	353 (5.7)	839 (13.5)	.9898	203 (3.3)	1322 (21.2)	.0023	955 (15.3)	741 (11.9)	.7958
Albuminuria	1 (0.0)	9 (0.1)	.2985	0 (0.0)	10 (0.2)	.3793	4 (0.1)	6 (0.1)	.3469
F/H of PCD	9 (0.1)	38 (0.6)	.1145	6 (0.1)	49 (0.8)	.3240	40 (0.6)	25 (0.4)	.4170

Note

Values are presented as *n* (%) unless specified otherwise.

Abbreviations: BMI, body mass index; CV, cardiovascular; F/H, family history; HbA1c, glycated haemoglobin; *N*, number of participants analysed; *n*, number of participants with non‐missing results at the visit; Ne, number of events; PCD, premature coronary disease

^a^ *p*‐Values are reported from Fisher's test if the cell frequency is lesser than 5. *p*‐values are reported using the *χ* ^2^ test otherwise. The null hypothesis is that there is no difference between the two population proportions. The *p*‐values reported are not adjusted for inflation in Type I error.

^b^ Percentages are calculated at baseline based on *N* = 3281 and at 1 year based on *N* = 3355.

^c^ Participants who had chosen ‘No’ and ‘Unknown’ for multiple complications are counted under ‘Unknown’.

### 3.4 Glycaemic status

At 1 year, all glycaemic parameters improved significantly from baseline (mean change: HbA1c: −0.5 [1.5] %; FPG: −12.2 [53.3] mg/dl; and PPG: −17.6 [76.5] mg/dl; *p* < .0001) (Figure 2). On HbA1c subgroup stratification, significant reduction in the number of participants was seen in the subgroups with HbA1c 8%–8.9% (*p* = .0028) and HbA1c ≥9% (*p* < .0001) with more participants entering the HbA1c 7%–7.9% (*p* < .0001) subgroup. However, only 20% of the participants achieved the optimum glycaemic control (HbA1c <7%) at 1 year (Figure S1). There was significant improvement (decrease) in the levels of glycaemic parameters at 1 year in insulin‐naïve as well as in subgroups receiving insulin (insulin, premix insulin and basal long‐acting insulin; *p* < .0001 for all except *p* = .0002 for PPG levels in the premix insulin subgroup) (Table 3). At 1 year, the number of participants with microvascular and macrovascular complications were more in those with HbA1c ≥7% than those with HbA1c <7% (Table S2).

**TABLE 3:** Comparison of glycaemic assessments between baseline and at 1 year by therapy group (*N* = 6236)

Therapy group	Glycaemic status	Baseline		1 year		Unadjusted *p*‐Value^a^
		*n*	Mean (95% CI)	*n*	Mean (95% CI)	
Insulin‐naïve at V1 to V3	HbA1c (%)	3027	7.7 (7.7, 7.8)	2875	7.4 (7.3, 7.4)	<.0001
	FPG (mg/dl)	3392	135.4 (134.0, 136.9)	3114	125.4 (124.3, 126.5)	<.0001
	PPG (mg/dl)	3322	194.3 (192.1, 196.5)	3053	179.9 (178.1, 181.6)	<.0001
Insulin at V1 to V3	HbA1c (%)	1036	8.7 (8.6, 8.8)	997	8.1 (8.0, 8.2)	<.0001
	FPG (mg/dl)	1164	155.4 (152.0, 158.8)	1169	137.6 (135.1, 140.1)	<.0001
	PPG (mg/dl)	1150	226.0 (221.3, 230.7)	1150	200.7 (197.2, 204.2)	<.0001
≤3 OADs at V1, V2 and V3 among insulin‐naïve	HbA1c (%)	1941	7.6 (7.6, 7.7)	1898	7.3 (7.3, 7.3)	<.0001
	FPG (mg/dl)	2161	133.6 (131.8, 135.5)	2075	123.2 (122.0, 124.4)	<.0001
	PPG (mg/dl)	2101	190.2 (187.5, 192.9)	2005	176.5 (174.5, 178.5)	<.0001
>3 OADs at V1, V2 and V3 among insulin‐naïve	HbA1c (%)	503	7.9 (7.7, 8.0)	496	7.5 (7.4, 7.6)	<.0001
	FPG (mg/dl)	577	137.4 (134.0, 140.8)	535	129.9 (126.8, 133.1)	.0027
	PPG (mg/dl)	564	199.3 (194.3, 204.3)	538	184.3 (179.8, 188.7)	<.0001
Basal long‐acting insulin at V1 to V3	HbA1c (%)	369	8.7 (8.5, 8.8)	372	8.0 (7.8, 8.1)	<.0001
	FPG (mg/dl)	405	153.5 (147.8, 159.3)	394	133.6 (129.6, 137.6)	<.0001
	PPG (mg/dl)	400	227.3 (218.9, 235.7)	375	192.0 (186.7, 197.3)	<.0001
Premix insulin at V1 to V3	HbA1c (%)	387	8.7 (8.5, 8.9)	353	8.1 (8.0, 8.3)	<.0001
	FPG (mg/dl)	440	155.7 (150.0, 161.5)	441	137.8 (133.8, 141.7)	<.0001
	PPG (mg/dl)	432	224.8 (217.3, 232.4)	437	204.7 (198.9, 210.5)	.0002

Abbreviations: CI, confidence interval; FPG, fasting plasma glucose; HbA1c, glycated haemoglobin; *n*, number of participants analysed; OAD, oral anti‐diabetic; PPG, postprandial glucose; V1, visit 1; V2, visit 2; V3, visit 3.

^a^ *p*‐Values are reported using a paired *t*‐test with the null hypothesis that the mean difference between the glycaemic status at baseline (V1) and at 1 year (V3) is equal. The *p*‐values reported are not adjusted for inflation in Type I error.

### 3.5 Vascular complications and glycaemic trends in metropolitan versus non‐metropolitan cities

The baseline age, duration and HbA1c parameters were comparable across participants of non‐metropolitan and metropolitan cities (Table S3A). The number of diabetes complications in metropolitan and non‐metropolitan cities increased over a period of 1 year. The microvascular complications, neuropathy, nephropathy and retinopathy were significantly higher in non‐metropolitan vs. metropolitan cities (*p* < .0001) (Table S3B). Among macrovascular complications, the number of participants with ACS was significantly higher in non‐metropolitan than in metropolitan cities (*p* < .05) (Table S3B).

At 1 year, a decrease was noted in all glycaemic parameters in both subgroups, in non‐metropolitan and metropolitan cities. However, the difference in fall of HbA1c from baseline between non‐metropolitan and metropolitan cities was not significant (mean [95% CI]: −0.5% [−0.5, −0.4] vs. −0.5% [−0.5, −0.4], *p* = .8613). Similarly, the difference in change from baseline for FPG and PPG between the non‐metropolitan and metropolitan cities was also not significant (*p* > .05) (Table S3A).

### 3.6 Anti‐diabetic treatment therapies

At 1 year, the proportion of participants taking OAD + insulin, increased (baseline: 1498/6236 [24.0%] vs. 1 year: 1844/6013 [30.7%]), while the proportion of those taking only OADs, decreased (baseline: 4642/6236 [74.4%] vs. 1 year: 4045/6013 [67.3%]) (Table S1). The number of participants receiving insulin or insulin along with OAD increased at 1 year with highest increase in the number of participants taking insulin with OAD was observed in the subgroup with diabetes for >10 years (OAD + insulin: baseline 690/1728 [39.9%] vs. 1 year 809/1667 [48.5%]) (Table S1). Biguanides and sulfonylureas were the most commonly prescribed OADs at baseline and at 1 year (biguanides, baseline: 5796/6236 [92.9%] and 1 year: 5620/6013 [93.5%]; sulfonylureas, baseline: 4758/6236 [76.3%] and 1 year: 4721/6013 [78.5%]). The highest increase in use was seen for dipeptidyl peptidase (DPP)‐IV inhibitors (baseline: 3047/6236 [48.9%] and 1 year: 3529/6013 [58.7%]) (Table S4). At 1 year, the commonly prescribed injectables were basal and premix insulins (basal insulin, baseline: 838/6236 [13.4%] and 1 year: 1130/6013 [18.8%]; premix insulin, baseline: 684/6236 [11.0%] and 1 year: 818/6013 [13.6%]) (Table S4).

Improvement in all glycaemic parameters at the end of 1 year was significantly higher in the insulin receiving subgroup than in the insulin‐naïve subgroup (*p* < .0001) (Table S5). Numerical decrease in levels of all glycaemic parameters (FPG, PPG and HbA1c) at 1 year was seen in both, basal long‐acting insulin and premix insulin subgroups. Among the insulin‐naïve participants, no statistical difference (*p* = .6872) was noted in the mean HbA1c values for those receiving >3 OADs vs. ≤3 OADs (Table S5).

### 3.7 Adverse drug reactions

A total of 13 events (12 participants) and 24 events (15 participants) of hypoglycaemia were recorded, during the first 6 months and the following 6 months of the 1‐year study period, respectively (Table S6). Four participants were hospitalized during the first 6 months of the initial year of the study due to myocardial infarction (two participants), ACS (one participant) and stroke (one participant). During the latter 6 months, one participant was hospitalized due to unstable angina (Table S6). No adverse drug reactions related to any Sanofi product were reported during the first year.

## 4 DISCUSSION

This article presents the real‐world trends observed in diabetes control and therapies in the LANDMARC study after the end of the first year, involving 6236 Indian adults with T2DM. The 1‐year data also provide a glimpse into the nature of the progression of vascular complications and accumulation of CV risk. At 1 year, only 20% participants achieved optimal glycaemic control (HbA1c <7%) and an increase was noted in the number of participants with diabetes complications. Hypertension and dyslipidaemia were the most common CV risk factors, observed more frequently in participants with BMI ≥23 kg/m^2^. Additionally, participants from metropolitan and non‐metropolitan cities showed similar decrease in glycaemic level; but microvascular complications (neuropathy, nephropathy and retinopathy) were significantly (*p* < .0001) higher in non‐metropolitan than metropolitan cities.

Hyperglycaemia leads to microvasculopathy and macrovasculopathy,^27^ but it is unclear whether the two vasculopathies progress concurrently or one precedes the other. Consistent optimal glycaemic control is vital in preventing or delaying diabetes complications. The United Kingdom Prospective Diabetes Study (UKPDS) observed that intense therapy for glycaemic control reduced the risk of myocardial infarction (15%; *p* = .01) and death from any cause (13%, *p* = .007) in the post‐trial period, while the reduction in the risk of microvascular disease persisted with enduring effects (24%; *p* = .001).^28^ Similarly, a meta‐analysis of four randomized controlled trials (ACCORD, ADVANCE, UKPDS and VADT) revealed that intensive glucose control compared with less intensive glucose control could reduce the relative risk for microvascular kidney events by 20% (*p* < .0001) and eye events by 13% (*p* = .04) but not for microvascular nerve events (hazard ratio: 0.98; *p* = .68).^29^ Diabetic neuropathy is prevalent in 10% of people at the time of T2DM diagnosis and in 40%–50% after 10 years of diagnosis^30^; it is even higher in those with poorer HbA1c control. Our study is in line with the existing literature. We observed a greater percentage of neuropathy cases among those with microvascular complications at 1 year. American Diabetes Association 2020 guidelines recommend optimizing glucose control to slow the progression of neuropathy in people with T2DM.^31^ The results obtained in our study indicate that occurrence of neuropathy is lower in participants with HbA1c <7% than in those with HbA1c ≥7%.

In this study, the burden of diabetes complications persisted and accumulated, and it was higher among those who were overweight, had suboptimal glycaemic control or displayed CV risk factors. Asians have a lower BMI but higher visceral fat than other ethnic groups, which makes them more susceptible to diabetes, high blood pressure and heart disease.^14^ Therefore, lower BMI cut‐offs are applied to classify overweight (BMI ≥23 kg/m^2^) and obese (BMI ≥25 kg/m^2^) categories for Indians.^32^ In the current study, 85% of the participants with T2DM had BMI ≥23 kg/m^2^ and were thus overweight or obese. A higher number of participants with BMI ≥23 kg/m^2^ reported hypertension or dyslipidaemia after a year than those with BMI <23 kg/m^2^. This underlines an urgent need to modulate treatment in people with T2DM targeting glycaemia and CV risk.

This longitudinal study revealed that the burden of uncontrolled diabetes is high in India with only 20% of participants achieving glycaemic control at 1 year (HbA1c <7%; 53 mmol/mol). This result indicates a lower percentage of glycaemic control (HbA1c ≤7% or <7%) than that observed in the Kerala study (HbA1c ≤7%; 28.3%), in the multicentric cross‐sectional ICMR‐INDIAB phase I study (HbA1c <7%; 31%) and that inferred from the 6 months data of the national diabetes registry covering 26 states across India (HbA1c <7%; 23.4%).^33^, ^34^, ^35^ The difference in the results could be attributed to the difference in the settings and designs among these studies. High levels of glycated haemoglobin persisting for more than 2 years can damage internal organs; a 2% decrease in HbA1c is required to prevent organ damage.^36^ Although the levels of the glycaemic indices in the present study improved marginally after 1 year, the mean values remained suboptimal (mean HbA1c 7.6%). The study results echo the progressive nature of diabetes that makes glycaemic control difficult over time as more than half of the participants in this study had diabetes for >5 years. An earlier Indian study revealed a significant increase in HbA1c levels corresponding to an increase in the duration of diabetes (0–1 year: 5.9 [2.2]%; 2–5 years: 7.9 [3.0]%; >5 years: 12.8 [2.4]%; *p* < .001).^37^

A large proportion of participants in our study were taking OADs alone at baseline (74.4%) as well as at 1 year (67.3%). Biguanides (93.5%) and sulfonylureas (78.5%) continued to be the most commonly reported OADs at the end of 1 year with highest increase observed in the use of DPP‐IV inhibitors (baseline: 3047 [48.9%]; 1 year: 3529 [58.7%]). Incretin‐based therapies, such as DPP‐IV inhibitors, are emerging as the preferred add‐on option to biguanides and/or SU, as observed from the 1‐year longitudinal trends, for T2DM because of their acceptable safety profile.^38^, ^39^

The TIGHT study, a retrospective analysis of 55,639 of Indian people with T2DM, reported that 86% participants were consuming dual or multiple anti‐diabetic drugs, which increased with the disease duration.^21^ Most of the participants who had diabetes for 2–10 years were predominantly only on OADs. There were participants in the study who were on >3 OADs even though guidelines recommend otherwise. There was overall no difference in the glucose‐lowering effects with a >3 OADs vs. those with ≤3 OADs in insulin‐naïve subgroup, thereby, indicating that there may be no additional glycaemic benefit in adding more than 3 OADs. Improvement in all glycaemic parameters was significantly higher in participants on insulin than in the insulin‐naïve subgroup (*p* < .0001). Early insulin initiation and/or timely intensification among people with uncontrolled T2DM would help to achieve rapid glycaemic control and, thereby, prevent the effects of prolonged glycaemic burden and slow down the disease progression.^40^ The findings of these studies may also indicate a clinical inertia prevalent in India related to diabetes management.

A previous study demonstrated that people living in metropolitan cities of India are at a high risk of developing diabetes which increases with an increase in BMI.^41^ Currently, there are no studies that provide information on the comparison between prevalence and disease management of people with T2DM in metropolitan vs. non‐metropolitan cities of India. As a step forward, the present data demonstrate that non‐metropolitan cities in India have a higher burden of diabetes complications, particularly microvascular. At 1 year, participants from both non‐metropolitan and metropolitan cities showed comparable improvement in glycaemic status with nearly identical trends in glycaemic targets. These findings complement the results of the previous study where good glycaemic control (HbA1c <7%) was observed in 30.8% of rural and 31.1% of urban participants.^34^ This is the first longitudinal trend data in India for non‐metropolitan vs. metropolitan cities, and thus may help to compare the longitudinal glycaemic patterns in India.

The strength of the study is its large sample size, representative of the T2DM population spread across India. The study design enabled us to capture a multitude of participant and disease characteristics, analyse trends influenced by known factors (BMI, sex, therapies), and identify new patterns (the number of OADs being used). Limitations of the study include missing values at 1 year, lack of data on factors such as socioeconomic status of the participants, smoking status and alcohol consumption. Another challenge in India is the economic burden of healthcare cost and accessibility to healthcare resources, which makes it difficult for some people who are diagnosed with diabetes to afford repetition of HbA1c test.

## 5 CONCLUSION

The 1‐year trend from the LANDMARC study highlights some important observations in terms of disease complications, treatment initiation and glycaemic control in the pan‐India cohort of participants with T2DM followed up in the real‐world setting. It indicates the need for early treatment initiation and its timely intensification to achieve and maintain the recommended glycaemic control, with an aim to reduce the persistent burden of microvascular and macrovascular complications. Apart from adopting effective treatment strategies, we believe that T2DM management can be achieved through increased disease awareness and focused education. Through this representative cohort, future long‐term follow‐up data would give us further insights about the development of complications, change in the treatment pattern and overall glycaemic status among participants' withT2DM in India.

## CONFLICT OF INTEREST

AKD, AM, AGU and NR received honoraria from Sanofi and other pharmaceutical companies. PKKM is on the advisory board of Sanofi and received honorarium for his talks. SJAQ6 received speaker/advisory/research grants from Abbott, Astrazeneca, Biocon, Boeringher Ingelheim, Eli Lilly, Franco Indian, Glenmark, Lupin, Marico, MSD, Novartis, Novo Nordisk, Roche, Sanofi, Serdia, Twinhealth and Zydus. SK received honoraria/speaker fees from Eli Lilly, Novo Nordisk and Sanofi. HT received honoraria from MSD, Novartis, Sanofi, and from other companies for advice and lectures. BS received honorarium from Aventis, Novo Nordisk, Eli Lilly, Boeringher‐Ingelham (BI) and MSD. **RG and SK were Sanofi employees; and AN, SM, SKM, DC, VS, ST and CT** are employees of Sanofi and may hold stock options. SC received honoraria/grants from Biocon, BI, Intas, Novartis, Sanofi, and Serdia. SKW has nothing to declare. AHZ received honoraria from Novo Nordisk, Eli Lilly, Johnson & Johnson, AstraZeneca, BI and Sanofi.

## AUTHOR CONTRIBUTIONS

**Ashok K. Das:** Conceptualization (equal); Data curation (supporting); Formal analysis (supporting); Funding acquisition (supporting); Investigation (lead); Methodology (equal); Project administration (supporting); Resources (supporting); Software (supporting); Supervision (lead); Validation (lead); Visualization (lead); Writing‐original draft (equal); Writing‐review & editing (equal). **Sanjay Kalra:** Conceptualization (supporting); Data curation (supporting); Formal analysis (supporting); Funding acquisition (supporting); Investigation (equal); Methodology (supporting); Project administration (supporting); Resources (supporting); Software (supporting); Supervision (equal); Validation (equal); Visualization (equal); Writing‐original draft (equal); Writing‐review & editing (equal). **Shashank Joshi:** Conceptualization (supporting); Data curation (supporting); Formal analysis (supporting); Funding acquisition (supporting); Investigation (equal); Methodology (supporting); Project administration (supporting); Resources (supporting); Software (supporting); Supervision (equal); Validation (equal); Visualization (equal); Writing‐original draft (equal); Writing‐review & editing (equal). **Ambrish Mithal:** Conceptualization (supporting); Data curation (supporting); Formal analysis (supporting); Funding acquisition (supporting); Investigation (equal); Methodology (supporting); Project administration (supporting); Resources (supporting); Software (supporting); Supervision (equal); Validation (equal); Visualization (equal); Writing‐original draft (equal); Writing‐review & editing (equal). **K. M. Prasanna Kumar:** Conceptualization (supporting); Data curation (supporting); Formal analysis (supporting); Funding acquisition (supporting); Investigation (equal); Methodology (supporting); Project administration (supporting); Resources (supporting); Software (supporting); Supervision (equal); Validation (equal); Visualization (equal); Writing‐original draft (equal); Writing‐review & editing (equal). **A. G. Unnikrishnan:** Conceptualization (supporting); Data curation (supporting); Formal analysis (supporting); Funding acquisition (supporting); Investigation (equal); Methodology (supporting); Project administration (supporting); Resources (supporting); Software (supporting); Supervision (equal); Validation (equal); Visualization (equal); Writing‐original draft (equal); Writing‐review & editing (equal). **Hemant Thacker:** Conceptualization (supporting); Data curation (supporting); Formal analysis (supporting); Funding acquisition (supporting); Investigation (equal); Methodology (supporting); Project administration (supporting); Resources (supporting); Software (supporting); Supervision (equal); Validation (equal); Visualization (equal); Writing‐original draft (equal); Writing‐review & editing (equal). **Bipin Sethi:** Conceptualization (supporting); Data curation (supporting); Formal analysis (supporting); Funding acquisition (supporting); Investigation (equal); Methodology (supporting); Project administration (supporting); Resources (supporting); Software (supporting); Supervision (equal); Validation (equal); Visualization (equal); Writing‐original draft (equal); Writing‐review & editing (equal). **Subhankar Chowdhury:** Conceptualization (supporting); Data curation (supporting); Formal analysis (supporting); Funding acquisition (supporting); Investigation (equal); Methodology (supporting); Project administration (supporting); Resources (supporting); Software (supporting); Supervision (equal); Validation (equal); Visualization (equal); Writing‐original draft (equal); Writing‐review & editing (equal). **Romik Ghosh:** Conceptualization (lead); Data curation (equal); Formal analysis (equal); Funding acquisition (lead); Investigation (equal); Methodology (lead); Project administration (lead); Resources (equal); Software (supporting); Supervision (equal); Validation (equal); Visualization (equal); Writing‐original draft (lead); Writing‐review & editing (lead). **Sukanya Krishnan:** Conceptualization (supporting); Data curation (lead); Formal analysis (equal); Funding acquisition (supporting); Investigation (equal); Methodology (equal); Project administration (equal); Resources (lead); Software (supporting); Supervision (equal); Validation (equal); Visualization (equal); Writing‐original draft (equal); Writing‐review & editing (equal). **Arjun Nair:** Conceptualization (supporting); Data curation (equal); Formal analysis (supporting); Funding acquisition (supporting); Investigation (equal); Methodology (equal); Project administration (supporting); Resources (supporting); Software (supporting); Supervision (equal); Validation (equal); Visualization (equal); Writing‐original draft (equal); Writing‐review & editing (equal). **Senthilnathan Mohanasundaram:** Conceptualization (equal); Data curation (equal); Formal analysis (supporting); Funding acquisition (equal); Investigation (equal); Methodology (equal); Project administration (equal); Resources (supporting); Software (supporting); Supervision (equal); Validation (equal); Visualization (equal); Writing‐original draft (equal); Writing‐review & editing (equal). **Shalini K. Menon:** Conceptualization (equal); Data curation (equal); Formal analysis (supporting); Funding acquisition (equal); Investigation (equal); Methodology (equal); Project administration (equal); Resources (supporting); Software (supporting); Supervision (equal); Validation (equal); Visualization (equal); Writing‐original draft (equal); Writing‐review & editing (equal). **Vaibhav Salvi:** Conceptualization (supporting); Data curation (equal); Formal analysis (equal); Funding acquisition (supporting); Investigation (equal); Methodology (equal); Project administration (equal); Resources (supporting); Software (equal); Supervision (equal); Validation (equal); Visualization (equal); Writing‐original draft (equal); Writing‐review & editing (equal). **Deepa Chodankar:** Conceptualization (equal); Data curation (equal); Formal analysis (lead); Funding acquisition (supporting); Investigation (equal); Methodology (lead); Project administration (equal); Resources (lead); Software (lead); Supervision (equal); Validation (equal); Visualization (equal); Writing‐original draft (equal); Writing‐review & editing (equal). **Saket Thaker:** Conceptualization (supporting); Data curation (supporting); Formal analysis (supporting); Funding acquisition (supporting); Investigation (equal); Methodology (supporting); Project administration (supporting); Resources (supporting); Software (supporting); Supervision (equal); Validation (equal); Visualization (equal); Writing‐original draft (equal); Writing‐review & editing (equal). **Chirag Trivedi:** Conceptualization (equal); Data curation (equal); Formal analysis (equal); Funding acquisition (equal); Investigation (equal); Methodology (equal); Project administration (equal); Resources (equal); Software (supporting); Supervision (equal); Validation (equal); Visualization (equal); Writing‐original draft (equal); Writing‐review & editing (equal). **Subhash K. Wangnoo:** Conceptualization (supporting); Data curation (supporting); Formal analysis (supporting); Funding acquisition (supporting); Investigation (equal); Methodology (supporting); Project administration (supporting); Resources (supporting); Software (supporting); Supervision (equal); Validation (equal); Visualization (equal); Writing‐original draft (equal); Writing‐review & editing (equal). **Abdul H. Zargar:** Conceptualization (supporting); Data curation (supporting); Formal analysis (supporting); Funding acquisition (supporting); Investigation (equal); Methodology (supporting); Project administration (supporting); Resources (supporting); Software (supporting); Supervision (equal); Validation (equal); Visualization (equal); Writing‐original draft (equal); Writing‐review & editing (equal). **Nadeem Rais:** Conceptualization (supporting); Data curation (supporting); Formal analysis (supporting); Funding acquisition (supporting); Investigation (equal); Methodology (supporting); Project administration (supporting); Resources (supporting); Software (supporting); Supervision (equal); Validation (equal); Visualization (equal); Writing‐original draft (equal); Writing‐review & editing (equal).

## Supporting information

Fig S1

Table S1‐S6

## Data Availability

Qualified researchers may request access to person‐level data and related study documents including the clinical study report, study protocol with any amendments, blank case report form, statistical analysis plan and data set specifications. Person‐level data will be anonymized, and study documents will be redacted to protect the privacy of trial participants. Further details on Sanofi's data sharing criteria, eligible studies and the process for requesting access can be found at https://www.clinicalstudydatarequest.com.
