# Supplementary figures and images for: One‐year trends from the LANDMARC trial: A 3‐year, pan‐India, prospective, longitudinal study on the management and real‐world outcomes of type 2 diabetes mellitus
Source: Endocrinol Diabetes Metab. 2021 Dec 1;5(1):e00316. doi: 10.1002/edm2.316 (PMC8754240; doi:10.1002/edm2.316)

**Baseline**

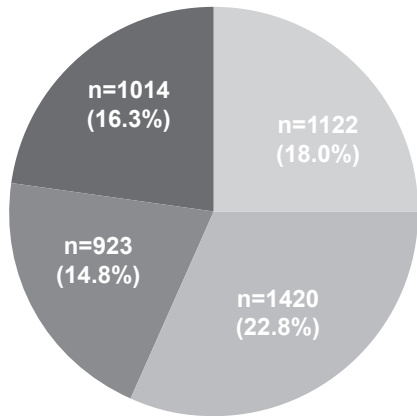

**HbA1c  
Categories**

- <7%
- 7-7.9%
- 8-8.9%
- ≥9%

**1-year**

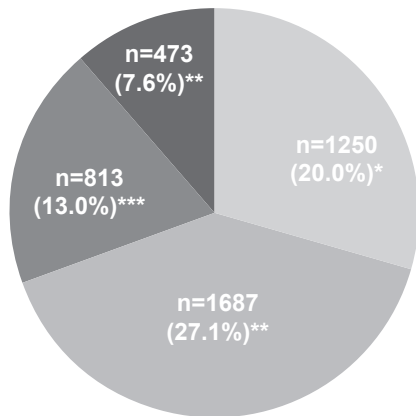

**HbA1c  
Categories**

- <7%
- 7-7.9%
- 8-8.9%
- ≥9%

Supplement: Supplementary file 1 — Fig S1 [file EDM2-5-e00316-s002.pdf]
