# Supplementary material for: One‐year trends from the LANDMARC trial: A 3‐year, pan‐India, prospective, longitudinal study on the management and real‐world outcomes of type 2 diabetes mellitus
Source: Endocrinol Diabetes Metab. 2021 Dec 1;5(1):e00316. doi: 10.1002/edm2.316 (PMC8754240; doi:10.1002/edm2.316)
Supplement: Supplementary file 2 — Table S1‐S6 [file EDM2-5-e00316-s001.docx]

**Table S1: Diabetes treatment stratified by diabetes duration at baseline and 1-year**

| **Duration of diabetes** | **2–5 years** | | **6-10 years** | | **>10 years** | | **Total** | |
| --- | --- | --- | --- | --- | --- | --- | --- | --- |
|  | **Baseline**  **N=2360**  **n (%)** | **1-year**  **N=2262**  **n (%)** | **Baseline**  **N=2148**  **n (%)** | **1-year**  **N=2084**  **n (%)** | **Baseline**  **N=1728**  **n (%)** | **1-year**  **N=1667**  **n (%)** | **Baseline**  **N=6236**  **n (%)** | **1-year**  **N=6013**  **n (%)** |
| Only on OAD | 2008 (85.1) | 1793 (79.3) | 1642 (76.4) | 1448 (69.5) | 992 (57.4) | 804 (48.2) | 4642 (74.4) | 4045 (67.3) |
| Only on Insulin | 8 (0.3) | 10 (0.4) | 5 (0.2) | 6 (0.3) | 13 (0.8) | 15 (0.9) | 26 (0.4) | 31 (0.5) |
| OAD + Insulin | 331 (14.0) | 441 (19.5) | 477 (22.2) | 594 (28.5) | 690 (39.9) | 809 (48.5) | 1498 (24.0) | 1844 (30.7) |
| OAD + non-insulin injectables | 11 (0.5) | 13 (0.6) | 16 (0.7) | 20 (1.0) | 18 (1.0) | 17 (1.0) | 45 (0.7) | 50 (0.8) |
| Insulin + non-insulin injectables | 0 | 0 | 0 | 1 (0.0) | 0 | 0 | 0 | 1 (0.0) |
| OAD + Insulin + non-insulin injectables | 2 (0.1) | 5 (0.2) | 8 (0.4) | 15 (0.7) | 15 (0.9) | 22 (1.3) | 25 (0.4) | 42 (0.7) |
| Values are presented as n (%) unless specified otherwise.  Note: Percentages are based on the number of participants using at least one anti-diabetic drug at the corresponding visit within each diabetes duration group. Total number of participants (N=6013) are the ongoing participants at 1 year.  N = number of participants analyzed; n = number of participants with non-missing results at the visit; OAD = oral anti-diabetic drugs. | | | | | | | | |

**Table S2: Microvascular and macrovascular complications at the end of first-year, by BMI, HbA1c and CV risk factors (N=6236)**

| **Complications** | **BMI <23 kg/m^2^ n (%)** | **BMI ≥23 kg/m^2^ n (%)** | **p-value*** | **HbA1c <7 % n (%)** | **HbA1c ≥7 % n (%)** | **p-value*** | **With CV risk factors n (%)** | **Without CV risk factors n (%)** | **p-value*** |
| --- | --- | --- | --- | --- | --- | --- | --- | --- | --- |
| Neuropathy | 108 (1.7) | 609 (9.8) | 0.6008 | 133 (2.1) | 380 (6.1) | 0.0518 | 590 (9.5) | 225 (3.6) | <0.0001 |
| Nephropathy | 22 (0.4) | 131 (2.1) | 0.6426 | 41 (0.7) | 80 (1.3) | 0.2948 | 144 (2.3) | 36 (0.6) | <0.0001 |
| Retinopathy | 31 (0.5) | 92 (1.5) | 0.0035 | 17 (0.3) | 78 (1.3) | 0.0115 | 117 (1.9) | 35 (0.6) | <0.0001 |
| MI | 0 | 4 (0.1) | >0.9999 | 1 (0.0) | 2 (0.0) | >0.9999 | 3 (0.1) | 1 (0.0) | 0.6287 |
| Stroke | 0 | 1 (0.0) | >0.9999 | 0 | 1 (0.0) | >0.9999 | 2 (0.0) | 0 | 0.5025 |
| PVD | 1 (0.0) | 9 (0.1) | >0.9999 | 1 (0.0) | 7 (0.1) | 0.4497 | 8 (0.1) | 3 (0.1) | 0.2394 |
| ACS | 2 (0.0) | 1 (0.0) | 0.0664 | 0 | 1 (0.0) | >0.9999 | 2 (0.0) | 1 (0.0) | >0.9999 |
| Values are presented as n (%) unless specified otherwise.  *****p-values are reported from Fisher’s test if the cell frequency is lesser than 5. p-values are reported using the Chi Square test otherwise. The null hypothesis is that there is no difference between the two population s’ proportions. The p-values reported are not adjusted for inflation in Type I error.  BMI = body mass index; HbA1c = glycated hemoglobin; ACS = acute coronary syndrome; CV = cardio-vascular; MI = myocardial infarction; N = number of participants analyzed; n = number of participants with non-missing results at the visit; PVD = peripheral vascular disease | | | | | | | | | |

**Table S3A: Participant characteristics and glycemic trends at the end of 1-year in metropolitan versus non-metropolitan cities**

| **Parameters** | | **Metropolitan cities**  **(N=2378)** | **Non-metropolitan cities**  **(N=3858)** |
| --- | --- | --- | --- |
| **Age (years)** | Mean (SD) | 52.4 (9.3) | 52.0 (9.0) |
| **Duration of T2DM (years)** | Mean (SD) | 8.62 (5.7) | 8.57 (5.6) |
| **Glycemic trends** | | | |
| **HbA1c (%)** | n | 1782 | 2441 |
|  | 1-year, mean (SD) | 7.6 (1.2) | 7.6 (1.2) |
|  | Change from baseline, mean (95% CI) | -0.5 (-0.5, -0.4) | -0.5 (-0.5, -0.4) |
|  | p value* | 0.8613 | |
| **FPG (mg/dL)** | n | 1862 | 2831 |
|  | 1-year, mean (SD) | 130.0 (37.2) | 129.4 (35.1) |
|  | Change from baseline, mean (95% CI) | -14.2 (-17.1, -11.2) | -11.2 (-13.3, -9.0) |
|  | p value* | 0.0966 | |
| **PPG (mg/dL)** | n | 1831 | 2786 |
|  | 1-year, mean (SD) | 183.9 (52.3) | 189.5 (55.9) |
|  | Change from baseline, mean (95% CI) | -21.4 (-25.5, -17.3) | -16.2 (-19.5, -13.0) |
|  | p value* | 0.0508 | |

**Table S3B: Prevalence of microvascular and macrovascular complications at the end of 1-year in metropolitan versus non-** **metropolitan cities**

| **Parameters** | | **Metropolitan cities**  **(N=2378)**  **n (%)** | **Non-metropolitan cities**  **(N=3858)**  **n (%)** |
| --- | --- | --- | --- |
| Neuropathy |  | 205 (8.6) | 610 (15.8)****** |
| Nephropathy |  | 36 (1.5) | 144 (3.7)****** |
| Retinopathy |  | 31 (1.3) | 121 (3.1)****** |
| MI† |  | 30 (1.3) | 48 (1.2) |
| Stroke† |  | 12 (0.5) | 20 (0.5) |
| PVD† |  | 21 (0.9) | 34 (0.9) |
| ACS‡ |  | 25 (1.1) | 70 (1.8)******* |

Metropolitan cities include Bengaluru, Chennai, Delhi, Hyderabad, Kolkata, and Mumbai.

Duration of T2DM (years) = Informed consent date − Start date of T2DM.

*****p-values are reported using an independent t-test with the null hypothesis that the mean change from baseline in glycemic status is equal in the two groups. The p-values reported are not adjusted for inflation in Type I error.

**p-values are reported from Fishers test if the cell frequency is lesser than 5. p-values are reported using the Chi Square test otherwise. The null hypothesis is that there is no difference between the two population proportions. The p-values reported are not adjusted for inflation in Type I error. **p<0.0001; ***p<0.05

†Events are part of the definition for the primary endpoint.

‡Events are part of the definition for the secondary endpoint.

Note: This is an interim analysis and possible modifications on variables and data could be performed for the subsequent interim analyses and the final analysis

ACS = acute coronary syndrome; CI = confidence interval; FPG = fasting plasma glucose; HbA1c = glycated hemoglobin; N = number of participants analyzed; n = number of participants with non-missing results; MI = myocardial infarction; PPG = postprandial glucose; PVD = peripheral vascular disease; SD = standard deviation; T2DM = type 2 diabetes mellitus.

**Table S4: Comparison of the use of oral and injectable glucose-lowering drugs at baseline and 1-year**

| **Anti-diabetic drug category** | **Baseline**  **N=6236**  **n (%)** | **1-year**  **N=6013**  **n (%)** |
| --- | --- | --- |
| **Oral anti-diabetic drugs** | 6210 (99.6) | 5981 (99.5) |
| Biguanides | 5796 (92.9) | 5620 (93.5) |
| Sulfonylureas | 4758 (76.3) | 4721 (78.5) |
| DPP-IV inhibitors | 3047 (48.9) | 3529 (58.7) |
| Thiazolidinediones | 697 (11.2) | 853 (14.2) |
| Alpha-glucosidase inhibitors | 1161 (18.6) | 1452 (24.1) |
| Meglitinides | 59 (0.9) | 70 (1.2) |
| Sodium glucose cotransporter 2 inhibitors | 654 (10.5) | 1013 (16.8) |
| Anti-malarial drug | 2 (0.0) | 4 (0.1) |
| **Injectable glucose lowering drugs** | 1594 (25.6) | 1968 (32.7) |
| GLP-1 analogs | 70 (1.1) | 93 (1.5) |
| Basal Insulin | 838 (13.4) | 1130 (18.8) |
| Prandial Insulin | 228 (3.7) | 301 (5.0) |
| Premix Insulin | 684 (11.0) | 818 (13.6) |
| Values are presented as n (%) unless specified otherwise.  DPP-IV = dipeptidyl peptidase-IV; GLP-1 = glucagon-like peptide-1; N = number of participants analyzed; n = number of participants with non-missing results at the visit | | |

**Table S5: Glycemic parameters according to treatment among people with type 2 diabetes mellitus from baseline to 1-year**

| **Therapy**  **Subgroup** | **HbA1c (%)  at 1-year** | | **Change from baseline  (%)** | | **p-value*** | **FPG (mg/dL)  at 1-year** | | **Change from baseline  (mg/dL)** | | **p-value*** | **PPG (mg/dL)  at 1-year** | | **Change from baseline  (mg/dL)** | | **p-value*** |
| --- | --- | --- | --- | --- | --- | --- | --- | --- | --- | --- | --- | --- | --- | --- | --- |
|  | **n** | **mean (95% CI)** | **n** | **mean (95% CI)** |  | **n** | **mean (95% CI)** | **n** | **mean (95% CI)** |  | **n** | **mean (95% CI)** | **n** | **mean (95% CI)** |  |
| **Insulin-naïve** | 2875 | 7.4 (7.3, 7.4) | 2294 | -0.3  (-0.4, -0.3) | <0.001 | 3114 | 125.4 (124.3, 126.5) | 2671 | -8.7  (-10.5, -7.0) | <0.0001 | 3053 | 179.9 (178.1, 181.6) | 2613 | -12.9  (-15.6, -10.3) | <0.0001 |
| **Insulin** | 997 | 8.1  (8.0, 8.2) | 805 | -0.7  (-0.8, -0.6) |  | 1169 | 137.6 (135.1, 140.1) | 995 | -16.4  (-20.4, -12.4) |  | 1150 | 200.7 (197.2, 204.2) | 980 | -24.3  (-29.8, -18.8) |  |
| **≤3 OADs among  insulin-naïve** | 1898 | 7.3 (7.3, 7.3) | 1509 | -0.3  (-0.4, -0.3) | 0.6380 | 2075 | 123.2 (122.0, 124.4) | 1770 | -9.2  (-11.2, -7.1) | 0.3577 | 2005 | 176.5 (174.5, 178.5) | 1711 | -13.5  (-16.6, -10.3) | 0.8685 |
| **>3 OADs among  insulin naïve** | 496 | 7.5 (7.4, 7.6) | 405 | -0.3  (-0.4, -0.2) |  | 535 | 129.9 (126.8, 133.1) | 469 | -7.0  (-11.6, -2.4) |  | 538 | 184.3 (179.8, 188.7) | 464 | -12.9  (-19.2, -6.5) |  |
| **Basal long acting insulin** | 372 | 8.0 (7.8, 8.1) | 297 | -0.9  (-1.1, -0.7) | 0.1502 | 394 | 133.6 (129.6, 137.6) | 335 | -18.2  (-24.7, -11.6) | 0.5546 | 375 | 192.0 (186.7, 197.3) | 331 | -32.9  (-41.7, -24.2) | 0.0314 |
| **Premix insulin** | 353 | 8.1 (8.0, 8.3) | 285 | -0.6  (-0.9, -0.4) |  | 441 | 137.8 (133.8, 141.7) | 381 | -15.3  (-22.1, -8.5) |  | 437 | 204.7 (198.9, 210.5) | 365 | -18.6  (-28.2, -9.0) |  |

* p-values are reported using an independent t-test with the null hypothesis that the mean change from baseline in glycemic status is equal in the two groups. The p-values reported are not adjusted for inflation in Type I error

HbA1c = glycated hemoglobin; FPG = fasting plasma glucose; OADs = oral anti-diabetics; PPG = postprandial glucose; n = number of participants with non-missing results at the visit

**Table S6: Hypoglycemic events and hospitalization**

|  | **Total N=6236** | | | |
| --- | --- | --- | --- | --- |
|  | **Baseline to 6-months** | | **6-month to 1-year** | |
|  | **n (%)** | **Ne** | **n (%)** | **Ne** |
| **Hypoglycemic events** | 12 (0.2) | 13 | 15 (0.2) | 24 |
| Severe hypoglycemia | 2 (0.0) | 2 | 1 (0.0) | 3 |
| Documented symptomatic hypoglycemia | 7 (0.1) | 7 | 9 (0.1) | 9 |
| Asymptomatic hypoglycemia | 3 (0.0) | 3 | 5 (0.1) | 7 |
| Nocturnal hypoglycemia | 1 (0.0) | 1 | 3 (0.0) | 5 |
| **Hospitalization** | 4 (0.1) | 4 | 1 (0.0) | 1 |
| Myocardial infarction | 2 (0.0) | 2 | 0 | 0 |
| Acute coronary syndrome | 1 (0.0) | 1 | 0 | 0 |
| Stroke | 1 (0.0) | 1 | 0 | 0 |
| Heart failure | 0 | 0 | 0 | 0 |
| Unstable angina | 0 | 0 | 1 (0.0) | 1 |
| Values are presented as n (%) unless specified otherwise.  Percentages are based on the number of participants within each subgroup in the eligible population. Eligible population includes all participants who have met inclusion/exclusion criteria for the study.  N = total number of participants analyzed; n = number of participants with hypoglycemic events/hospitalization; Ne = number of hypoglycemic/hospitalization events | | | | |

# Appendix 2: Supporting Figure

**Figure S1: Proportion of participants across HbA1c categories (N=6236)**

Data presented as n (%) from baseline (N=6236). HbA1c was not measured for all participants and hence the percentage may not add up to 100%.

p-values are reported using McNemar’s test with the null hypothesis that the proportion of paired samples are equal. Participants who met each criteria and those who did not meet the criteria are considered as binary outcomes for the test. The p-values reported are not adjusted for inflation in Type I error.

*p=0.0006; **p<0.0001; ***p=0.0028

HbA1c = glycated hemoglobin; N = number of participants analyzed; n = number of participants with non-missing results at the visit
